# Effects of exercise therapy on patients with poststroke cognitive impairment: A systematic review and meta-analysis

**DOI:** 10.3389/fnins.2023.1164192

**Published:** 2023-04-06

**Authors:** Yuanxing Zhang, Xichenhui Qiu, Jinghao Chen, Cuiling Ji, Fang Wang, Dan Song, Caiyan Liu, Lu Chen, Ping Yuan

**Affiliations:** ^1^Department of Neurosurgery, Nanjing Drum Tower Hospital Clinical College of Nanjing University of Chinese Medicine, Nanjing, China; ^2^Health Science Center, Shenzhen University, Shenzhen, China; ^3^Department of Neurosurgery, Nanjing Drum Tower Hospital, The Affiliated Hospital of Nanjing University Medical School, Nanjing, Jiangsu Province, China; ^4^Nursing Department, Shenzhen Shekou People's Hospital, Shenzhen, China

**Keywords:** stroke, cognitive impairment, exercise therapy, systematic review, meta-analysis

## Abstract

**Objective:**

To evaluate the effects of exercise therapy on patients with poststroke cognitive impairment and compare the differences in the effect of this method when compared with conventional measures, providing evidence for a more standardized and effective clinical application of exercise therapy.

**Methods:**

A search was conducted using 7 electronic databases, including PubMed, CINAHL, Web of Science, CENTRAL, CNKI, Wanfang, SinoMed, and clinical trials registry platforms for randomized controlled trials concerning exercise therapy on patients with poststroke cognitive impairment. Two researchers independently screened the literature, evaluated the quality, and extracted information. Meta-analysis was carried out using Review Manager 5.4 software.

**Results:**

There were 11 studies with 1,382 patients. Meta-analysis showed that exercise therapy could improve cognitive function [*SMD* = 0.67, 95% CI (0.31, 1.04), *P* = 0.0003], motor function [*SMD* = 1.81, 95% CI (0.41, 3.20), *P* = 0.01], and the activities of daily living [*MD* = 8.11, 95% CI (3.07, 13.16), *P* = 0.002] in patients with poststroke cognitive impairment.

**Conclusion:**

Exercise therapy can not only improve cognitive function in patients with poststroke cognitive impairment but also improve motor function and the activities of daily living. Medical staff should prioritize the management of patients with poststroke cognitive impairment and carry out exercise therapy actively to improve the cognitive function of patients with stroke.

**Systematic review registration:**

https://www.crd.york.ac.uk/prospero/, identifier: CRD42023397553.

## Introduction

Stroke is an important cause of cognitive impairment and dementia (Zhao et al., 2018). Poststroke cognitive impairment (PSCI) refers to cognitive impairment or dementia after a stroke. It is common in patients with stroke and usually occurs within 6 months after the stroke. The prevalence of PSCI ranges from 20 to 80% and is one of the most common complications in patients with stroke (Sun et al., 2014). PSCI is an important factor that seriously affects patients' quality of life and survival time, and it has evolved into one of the hot topics in stroke research and intervention (Dong et al., 2017). Due to cognitive impairment, patients' cognitive abilities decline, and their adaptability to the external environment is disturbed. Therefore, patients are prone to emotional disorders such as anxiety and depression. It can also be characterized by impaired memory function, decreased computing power, and abstract thinking, which affects not only the daily lives of patients but also their rehabilitation of limbs and neurological functions. It can induce a secondary stroke and even threaten their lives, seriously affecting the overall rehabilitation process (Dong et al., 2021). Stroke survivors with moderate PSCI were six times more likely to progress to occasional dementia than stroke survivors without cognitive impairment, and up to 25% of patients with cognitive impairment were diagnosed with dementia within 3 years of stroke (Narasimhalu et al., 2009; Sachdev et al., 2009). Therefore, the rehabilitation of cognitive function in patients with stroke is an urgent issue.

Early intervention is particularly important for patients with PSCI. Studies have shown that there is a wide variation in the treatment of cognitive problems after stroke, including pharmacological and non-pharmacological interventions (Quinn et al., 2021). However, the long-term efficacy of pharmacological interventions is unclear and may be associated with adverse effects. For example, an analysis of the evidence suggests that actovegin and cerebrolysin are animal-derived nootropics that may have potential efficacy in the treatment of neurodegenerative diseases. It has a beneficial effect on improving cognitive function after stroke (Quinn et al., 2021). However, the most common adverse event was a recurrent ischemic stroke. Therefore, more researchers are inclined toward non-pharmacological interventions, such as exercise therapy, cognitive intervention, and acupuncture therapy. Early non-pharmacological exercise therapy for patients can delay the progression of the disease, sometimes even reverse the process of cognitive decline, and reduce the disability rate. Huang et al. (2022) conducted a network meta-analysis of the comparative effectiveness of different exercise interventions on cognitive function in patients with mild cognitive impairment or dementia and found that all types of exercise can effectively improve overall cognitive function in patients. However, there is a lack of effective evidence for exercise therapy in patients with PSCI.

Although routine rehabilitation training can delay the process of cognitive decline in patients and prevent the disease from progressing to dementia, there are shortcomings, such as a single form of training, low patient acceptance, and difficulty in conducting continuous and effective training, which are not conducive to the recovery of cognitive function (Yu et al., 2019). Some studies have found that exercise therapy can improve health by increasing oxygen and blood supply to the brain and indirectly improving cognitive impairment (Tang et al., 2020). Exercise therapy is defined as “a regimen or plan of physical activity designed and prescribed for specific therapeutic goals with the purpose of restoring normal physical function or reducing symptoms caused by disease or injury (Caspersen et al., 1985)”. The regimen includes aerobic exercise, resistance exercise, and multiple combination exercises, as well as some traditional Chinese medicine exercises such as Baduanjin. Traditional Chinese medicine exercise therapy has been found to improve cognitive function in elderly patients with mild cognitive impairment (MCI) by regulating cognition-related brain function and structure (Su et al., 2022). Although there are many studies on the use of exercise therapy to improve PSCI, there is a relative lack of consensus, and there is no meta-analysis on the effects of exercise therapy on patients with PSCI. This study evaluated the effects of exercise therapy on patients with PSCI through a meta-analysis, aiming to provide a new evidence-based basis for intervention in patients with PSCI.

This systematic evaluation program is registered in the PROSPERO database (CRD42023397553).

## Materials and methods

### Search strategy

Two researchers searched PubMed, CINAHL, Web of Science, Cochrane Central Register of Controlled Trials (CENTRAL), China National Knowledge Infrastructure (CNKI), Wanfang Database, Chinese Biomedical Literature Service System (SinoMed), and the clinical trials registry platform. The search was conducted from the database creation date to January 2023. In addition, the search was conducted by combining free words and subject terms. The search formula was (stroke OR cerebrovascular OR hemiplegia OR cerebral hemorrhage OR cerebral infarction OR cerebral stroke OR acute stroke) AND (cognitive dysfunction OR cognitive impairment OR cognition disorders) AND (physical activity OR physiotherapy OR fitness OR aerobic OR exercise OR resistance training OR physical fitness OR exercise). We searched both the included references and the gray literature. The results were cross-checked after each of the two researchers had completed the search independently. In case of disagreement, the decision was discussed with a third researcher.

### Study design and eligibility criteria

This systematic review was completed according to the Cochrane Collaboration methodology and the Preferred Reporting Items for Systematic Reviews and Meta-Analyses (PRISMA) checklist (Sachdev et al., 2009).

A participant-intervention-comparison-outcome (PICO) strategy was used to structure the research questions (Stone, 2002). The inclusion criteria were as follows: (1) Participants: patients who meet the diagnostic criteria adopted by the Fourth National Conference on Cerebrovascular Diseases and were diagnosed with ischemic or hemorrhagic stroke by CT or MRI examination, are over 18 years of age, and had a cognitive decline occurring within 6 months of stroke; (2) Intervention: exercise therapy, including aerobic exercise, resistance exercise, and multiple combination exercises; (3) Comparison: routine non-pharmacological intervention, including a balanced diet, health education, and routine rehabilitation training; (4) Outcome: the main outcome indicator was cognitive function, and the assessment tools were the Minimum Mental State Examination (MMSE) and the Montreal Cognitive Assessment (MoCA), and the secondary outcome indicators were motor function and activities of daily living, measured by the Fugl-Meyer Assessment (FMA) and the Modified Barthel Index (MBI). In addition, the study design must be a randomized controlled trial (RCT). The exclusion criteria were as follows: (1) data could not be extracted; (2) the full text could not be obtained; (3) the literature is a repeated publication; (4) the literature is a conference paper; and (5) the literature quality assessment was high risk.

### Data extraction

Two researchers trained in evidence-based research independently searched the literature, imported the retrieved literature into the Endnote software, and deleted duplicate literature; they simultaneously and independently read the titles and abstracts for preliminary screening and carefully read the full text to determine the included literature according to the inclusion and exclusion criteria. The two researchers extracted information from the literature, including the year of publication, country, sample size, intervention measures of the experimental and control groups, intervention duration, outcome indicator, and evaluation tool. In case of disagreement, the decision was discussed with a third researcher. The researchers contacted the author by phone or email to request additional information.

### Quality appraisal

The quality of included RCTs was assessed independently by two reviewers using the Cochrane Systematic Review Manual 5.1.0, with a third researcher consulted to reach a consensus in case of disagreement. The evaluation included (1) random sequence generation (selection bias), (2) allocation concealment (selection bias), (3) blinding of participants and personnel (performance bias), (4) blinding of outcome assessment (detection bias), (5) incomplete outcome data (attrition bias), (6) selective reporting (reporting bias), and (7) other biases. The bias for the abovementioned 7 aspects is low bias, high bias, and unclear (lack of relevant information or bias situation is uncertain).

### Statistical analysis

The RevMan software (version 5.4; Cochrane Collaboration, Copenhagen, Denmark) was used for meta-analysis. Based on the type of extracted data, we evaluated 95% confidence intervals (CIs) for continuous variables. For a continuous variable that was measured using different scales, we used standardized mean differences (SMD) as a measure for effect size; for a continuous variable that was measured using the same scale, we used mean differences (MD) for effect size. A *P*-value of < 0.05 (two-sided) was considered statistically significant in the estimation of effects. *I*^2^ was used to determine the heterogeneity of the results. If the *P-*value was > 0.1 and *I*^2^ was < 50%, indicating low heterogeneity, and if all studies were from a homogeneous population, the fixed-effects model was used for meta-analysis. If the *P-*value was ≤ 0.1 and *I*^2^ was ≥ 50%, indicating large heterogeneity, the source of heterogeneity was analyzed as far as possible. If the heterogeneity could not be reduced, the random-effects model was used for the meta-analysis. The inverse variance method was used to pool the effect-size measure. Clinical and methodological heterogeneity was addressed by sensitivity analysis, subgroup analysis, or descriptive analysis only.

## Results

### Study selection

**Figure 1:**
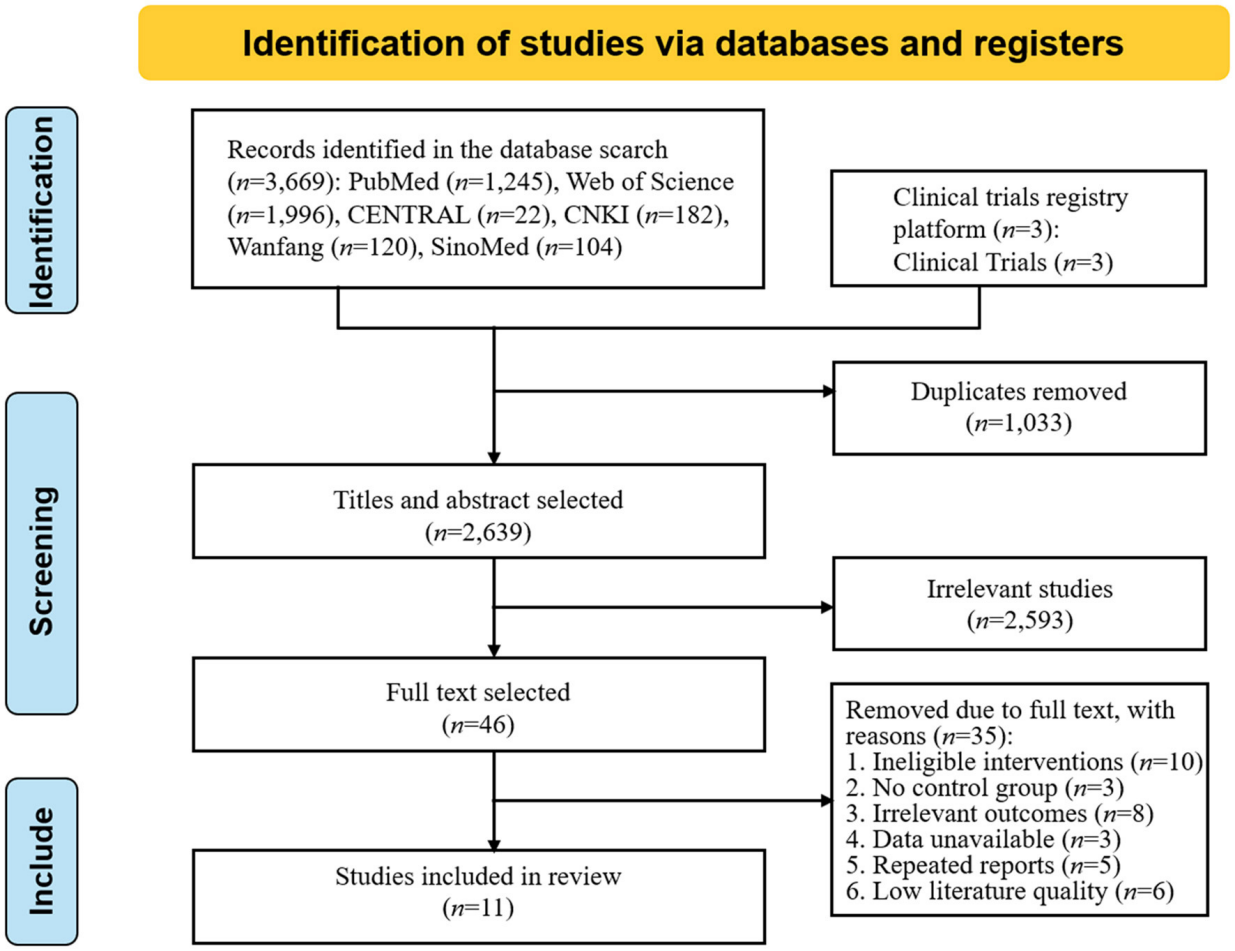
Screening process of literature selection.

According to the search strategy, 3,672 studies were preliminarily searched, 1,033 duplicate studies were excluded, and 11 studies (Fang et al., 2003; Studenski et al., 2005; El-Tamawy et al., 2014; Zhang et al., 2015, 2020; Fernandez-Gonzalo et al., 2016; Kim and Yim, 2017; Li, 2017; Ihle-Hansen et al., 2019; Yu et al., 2019; Zheng et al., 2020) were finally included after the preliminary screening of the title abstract and reading the full text. The literature screening process and results are shown in Figure 1.

### Characteristics of the included studies

A total of 11 studies were included in this review, and the characteristics of each study are shown in Table 1. Only Chinese and English literature were included in this review, and literature in other languages was not considered. The sample sizes of the RCTs ranged from 20 to 420 participants. The total sample size for the meta-analysis was 1,382 participants, including both experimental (*n* = 679) and control (*n* = 703) participants.

**Table 1:** Characteristics of included studies in this review.

**References**	**Country**	**Sample size (experiment group/control group)**	**Intervention**		**Intervention duration**	**Outcome indicator**	**Evaluation tool**
			**Experiment group**	**Control group**			
Fang et al. (2003)	China	50/78	Early physiotherapy (Bobath techniques and passive movements training of the affected limb during the first week of stroke onset); 45 min/day, 5 days/week	Routine therapy without early professional physiotherapy	4 weeks	Cognitive function, motor function, activities of daily living	MMSE, FMA, MBI
Ihle-Hansen et al. (2019)	Norway	143/156	Physical activity 30 min/day, physical exercise 45 to 60 min/week, including at least 2-3 vigorous exercise	Conventional rehabilitation	18 months	Cognitive function	MMSE
Kim and Yim (2017)	South Korea	14/15	Exercise protocol for handgrip strength (grasping training with a power web exerciser + Digi-Flex repetition training) and walking speed (run on a motor-powered treadmill) for 30 min, 3 times per day	Conventional physical therapy for 60 min per day	6 weeks	Cognitive function, motor function	MoCA, Timed Up and Go
Li (2017)	China	42/42	Aerobic combined with resistance exercise, including aerobics 40 min/time, 5 times/week, balance training and use of resistance equipment for resistance training 20 min/time, 3 times/week	Routine nursing procedure	3 months	Cognitive function, activities of daily living	MMSE, MBI
El-Tamawy et al. (2014)	Egypt	15/15	Cardio on a bicycle dynamometer for “40–45” min, 3 times/week	Physiotherapy program “25–30” min/session, 3 times/week	8 weeks	Cognitive function	ACER
Fernandez-Gonzalo et al. (2016)	Switzerland	10/10	Lower extremity resistance training (using the more-affected limb, 4 sets of 7 repetitions; < 2 min of contractile activity) 2 times/week	Daily routine rehabilitation training	12 weeks	Cognitive function	TMT B
Studenski et al. (2005)	USA	44/49	Home-based exercise program, supervised by an occupational or physical therapist (major muscle groups of the upper and lower extremity using elastic bands and body weight, using an exercise bicycle)	Conventional rehabilitation (health education, vital signs, and an oxygen saturation test)	12 weeks	Cognitive function, motor function, activities of daily living	FIM, MBI
Yu et al. (2019)	China	50/50	Aerobic exercise combined with resistance exercise (health care relaxation exercise and acupoint massage 40 min/time, 4 times/week +upper and lower limb exercises using elastic bands, 20 min/time, 4 times/week	Routine rehabilitation training and health education	None	Cognitive function, motor function	MoCA, FMA
Zhang et al. (2015)	China	220/200	Aerobic combined with resistance exercise (aerobics 40 min/time, 5 times/week, balance training and resistance training 20 min/time, 3 times/week	Health education and routine rehabilitation training	None	Cognitive function	MoCA
Zhang et al. (2020)	China	69/69	Indoor bicycle aerobic training, 20–30 min/ time, 4 times/week	Routine neuromuscular rehabilitation therapy and health education	12 weeks	Cognitive function, motor function, activities of daily living	MMSE, FMA, MBI
Zheng et al. (2020)	China	22/19	Baduanjin training 40 min/day, 3 days/week	Routine rehabilitative treatment and health education	24 weeks	Cognitive function, activities of daily living	MoCA, MBI

### Quality assessment

**Figure 2:**
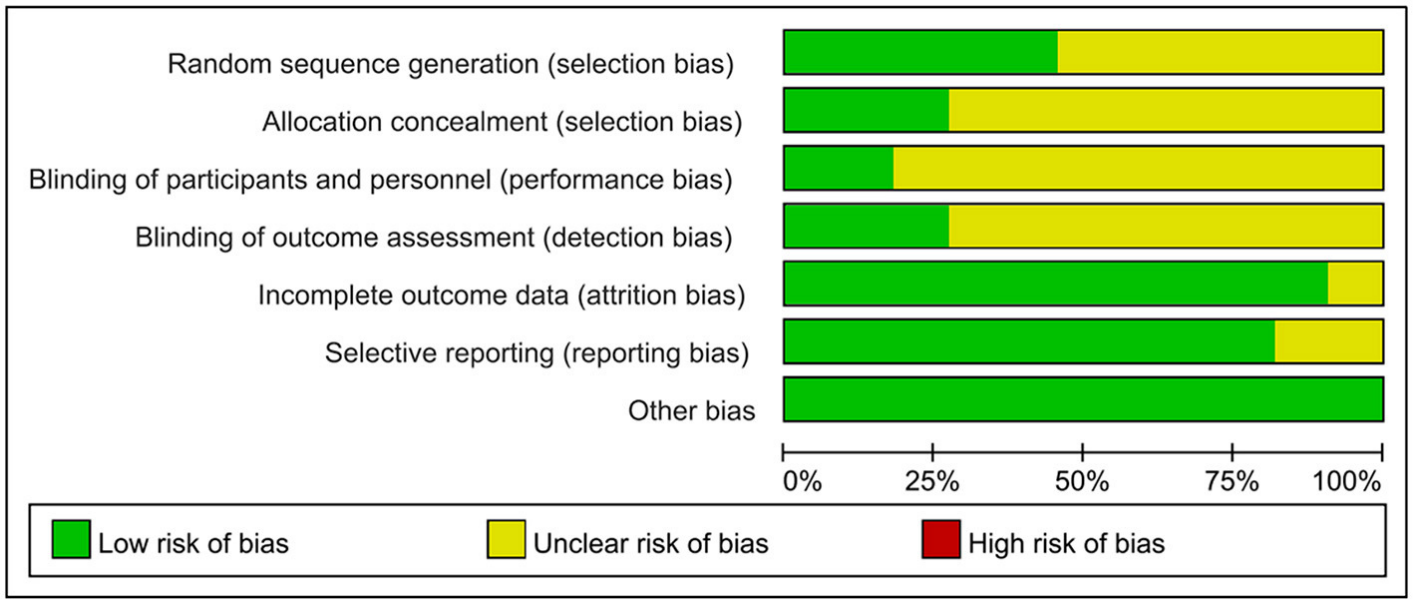
Risk assessment of bias.

The 11 studies included in this analysis were RCTs, all of which had clearly defined inclusion and exclusion criteria for subjects and were comparable at baseline. The tools used to measure outcomes in the experimental and control groups were consistent, and the same statistical methods were used. The method of random sequence generation was explained in 5 studies (Fang et al., 2003; Zhang et al., 2015, 2020; Ihle-Hansen et al., 2019; Zheng et al., 2020), and allocation concealment was described in detail in only 3 studies (Fang et al., 2003; Ihle-Hansen et al., 2019; Zheng et al., 2020). The blinding of outcome evaluators was explicitly described in 3 studies (Fang et al., 2003; Studenski et al., 2005; Zheng et al., 2020), and the blinding of subjects and intervention implementers was explicitly described in 2 studies (Studenski et al., 2005; Zheng et al., 2020). Only 1 study (Fang et al., 2003) had missing data, and it did not explain the method of handling missing data; no other sources of bias were found in all studies. The literature quality assessment is shown in Table 2, Figure 2.

**Table 2:** Quality evaluation of the included literature.

**References**	**Random sequence generation**	**Allocation concealment**	**Blinding of participants and personnel**	**Blinding of outcome assessment**	**Incomplete outcome data**	**Selective reporting**	**Other bias**
Fang et al. (2003)	Low risk	Low risk	Unclear	Low risk	Unclear	Unclear	Low risk
Ihle-Hansen et al. (2019)	Low risk	Low risk	Unclear	Unclear	Low risk	Low risk	Low risk
Kim and Yim (2017)	Unclear	Unclear	Unclear	Unclear	Low risk	Low risk	Low risk
Li (2017)	Unclear	Unclear	Unclear	Unclear	Low risk	Low risk	Low risk
El-Tamawy et al. (2014)	Unclear	Unclear	Unclear	Unclear	Low risk	Low risk	Low risk
Fernandez-Gonzalo et al. (2016)	Unclear	Unclear	Unclear	Unclear	Low risk	Unclear	Low risk
Studenski et al. (2005)	Unclear	Unclear	Low risk	Low risk	Low risk	Low risk	Low risk
Yu et al. (2019)	Unclear	Unclear	Unclear	Unclear	Low risk	Low risk	Low risk
Zhang et al. (2015)	Low risk	Unclear	Unclear	Unclear	Low risk	Low risk	Low risk
Zhang et al. (2020)	Low risk	Unclear	Unclear	Unclear	Low risk	Low risk	Low risk
Zheng et al. (2020)	Low risk	Low risk	Low risk	Low risk	Low risk	Low risk	Low risk

## Results of meta-analysis

### Effects of exercise therapy on cognitive function in patients with PSCI

**Figure 3:**
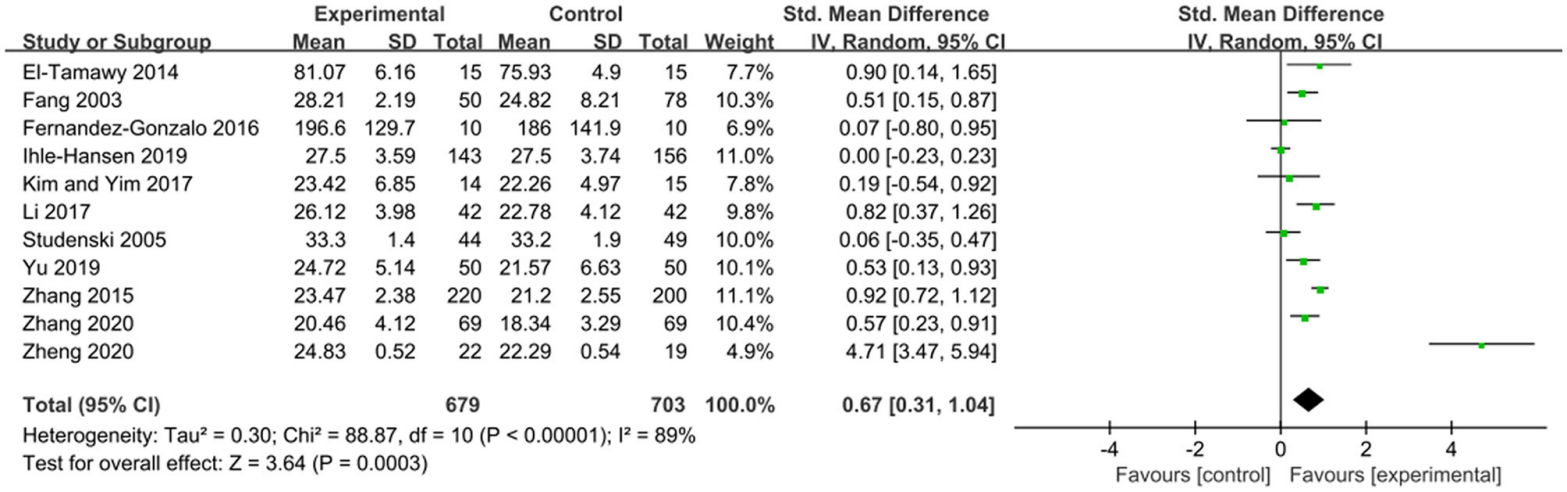
Effects of exercise therapy on cognitive function in patients with PSCI.

The MMSE scale and the MoCA scale are currently widely used cognitive function assessment scales in clinical practice, both of which can be used to assess patients' cognitive function (Jia et al., 2021). Both of them are effective in diagnosing dementia and cognitive impairment (Pinto et al., 2019). The effects of exercise therapy on cognitive function in patients with PSCI were reported in 11 studies (Fang et al., 2003; Studenski et al., 2005; El-Tamawy et al., 2014; Zhang et al., 2015, 2020; Fernandez-Gonzalo et al., 2016; Kim and Yim, 2017; Li, 2017; Ihle-Hansen et al., 2019; Yu et al., 2019; Zheng et al., 2020). *SMD* was selected for the combination of effect sizes, and the results showed significant heterogeneity (*P* < 0.01, *I*^2^ = 89%). When the study by Zheng et al. (2020) was removed using sensitivity analysis, the heterogeneity was reduced, and the 95% CI was (0.20, 0.74), but *I*^2^ was equal to 80%. The random-effects model was used for the meta-analysis. The results showed that the cognitive function of the experimental group was higher than that of the control group after the intervention, and the difference was statistically significant [*SMD* = 0.67, 95% CI (0.31, 1.04), *P* = 0.0003], as shown in Figure 3.

**Figure 4:**
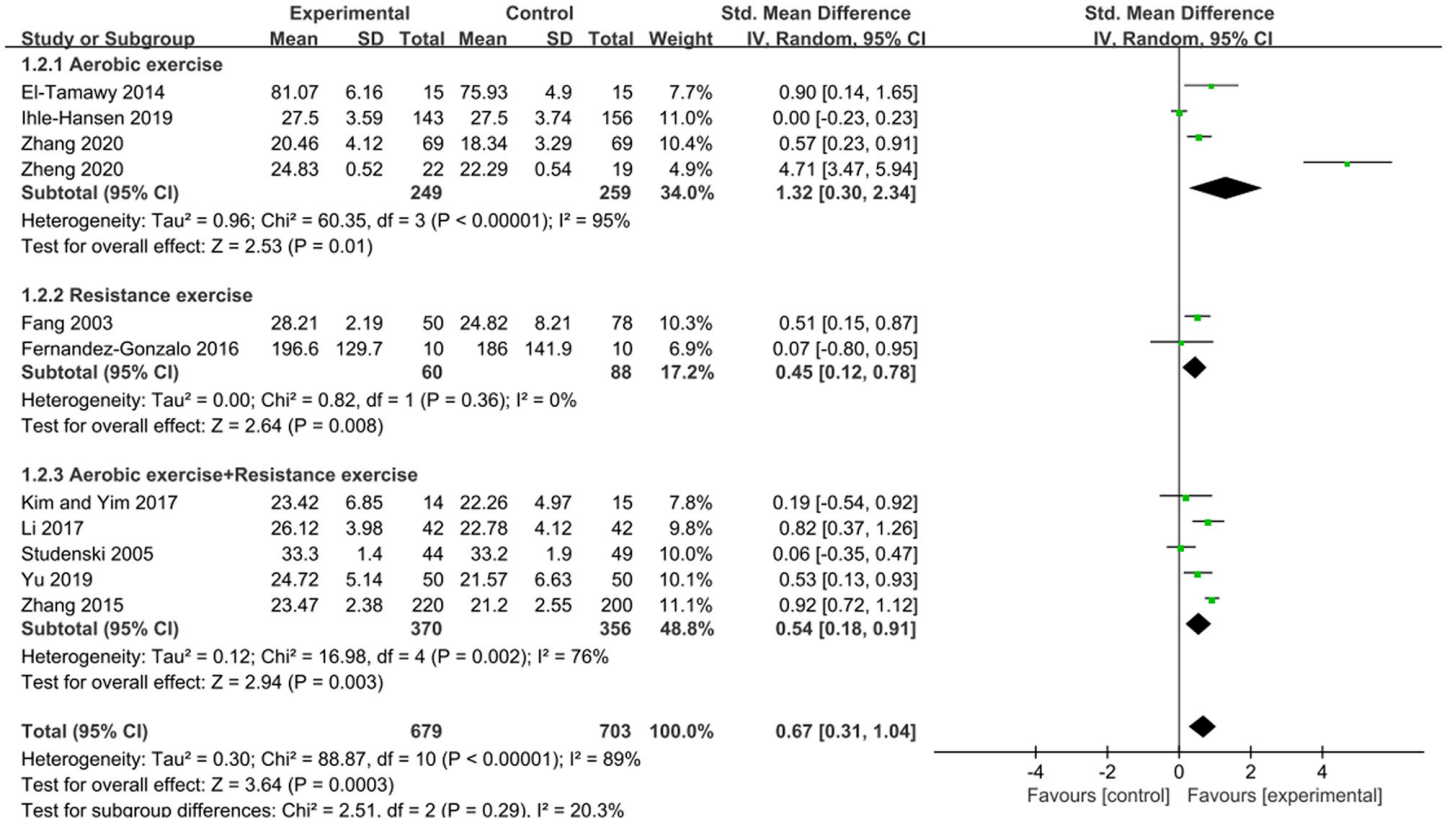
Subgroup analysis of the effects of different types of exercise on cognitive function in patients with PSCI.

Subgroup analysis was performed based on the type of exercise: aerobic exercise was used in 4 studies (El-Tamawy et al., 2014; Ihle-Hansen et al., 2019; Zhang et al., 2020; Zheng et al., 2020); resistance exercise was used in 2 studies (Fang et al., 2003; Fernandez-Gonzalo et al., 2016); and aerobic exercise combined with resistance exercise was used in 5 studies (Studenski et al., 2005; Zhang et al., 2015; Kim and Yim, 2017; Li, 2017; Yu et al., 2019). In subgroup analysis (as shown in Figure 4), we found that each exercise type had a positive impact on cognitive function, with a *p*-value of < 0.05 for the overall effect for each exercise type. Moreover, aerobic exercise showed a large clinical effect in improving cognitive function [*SMD* = 1.32, 95%CI (0.30, 2.34)], while the other two exercise types exhibited a medium effect (*SMD* ≈ 0.5). However, the differences were not statistically significant (*P* = 0.29 from the test for subgroup differences) (Andrade, 2020).

### Effects of exercise therapy on motor function in patients with PSCI

**Figure 5:**
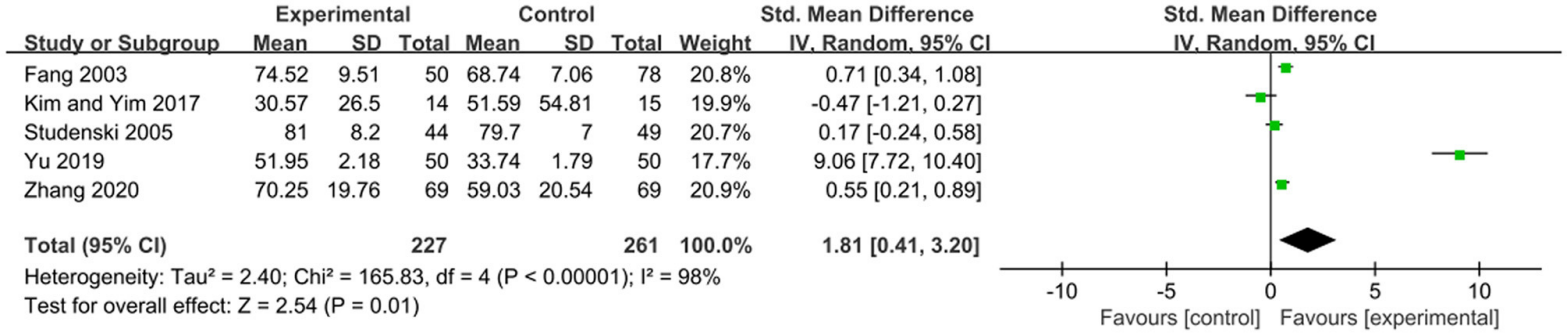
Effects of exercise therapy on monitor function in patients with PSCI.

The FMA scale is considered by many in the field of stroke rehabilitation as one of the most comprehensive quantitative measures of motor impairment after stroke (Gladstone et al., 2002). The effects of exercise therapy on motor function in patients with PSCI were reported in 5 studies (Fang et al., 2003; Studenski et al., 2005; Kim and Yim, 2017; Yu et al., 2019; Zhang et al., 2020). *SMD* was selected for the combination of effect sizes, and the results showed significant heterogeneity (*P* < 0.01, *I*^2^ = 98%). When the study by Yu et al. (2019) was removed using sensitivity analysis, the heterogeneity was reduced, and the 95% CI was (−0.06, 0.72) but *I*^2^ was equal to 70%. The random-effects model was used for the meta-analysis. The results showed that the motor function of the experimental group was higher than that of the control group after the intervention, and the difference was statistically significant [*SMD* = 1.81, 95% CI (0.41, 3.20), *P* = 0.01], as shown in Figure 5.

### Effects of exercise therapy on activities of daily living in patients with PSCI

**Figure 6:**
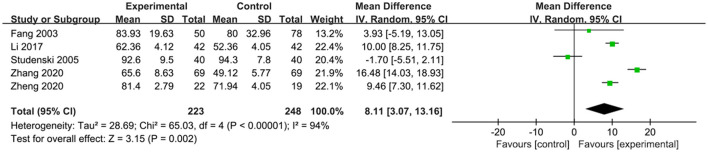
Effects of exercise therapy on activities of daily living in patients with PSCI.

The effects of exercise therapy on the activities of daily living in patients with PSCI were reported in 5 studies (Fang et al., 2003; Studenski et al., 2005; Li, 2017; Zhang et al., 2020; Zheng et al., 2020). *SMD* was selected for the combination of effect sizes, and the results showed significant heterogeneity (P < 0.01, *I*^2^ = 94%). When the study by Studenski et al. (2005) was removed using sensitivity analysis, heterogeneity was reduced, and the 95% CI was (7.14, 14.86), but *I*^2^ was equal to 88%. The random-effects model was used for the meta-analysis. The results showed that the activities of daily living of the experimental group were higher than those of the control group after the intervention, and the difference was statistically significant [*MD* = 8.11, 95% CI (3.07, 13.16), *P* = 0.002], as shown in Figure 6.

### Publication bias

**Figure 7:**
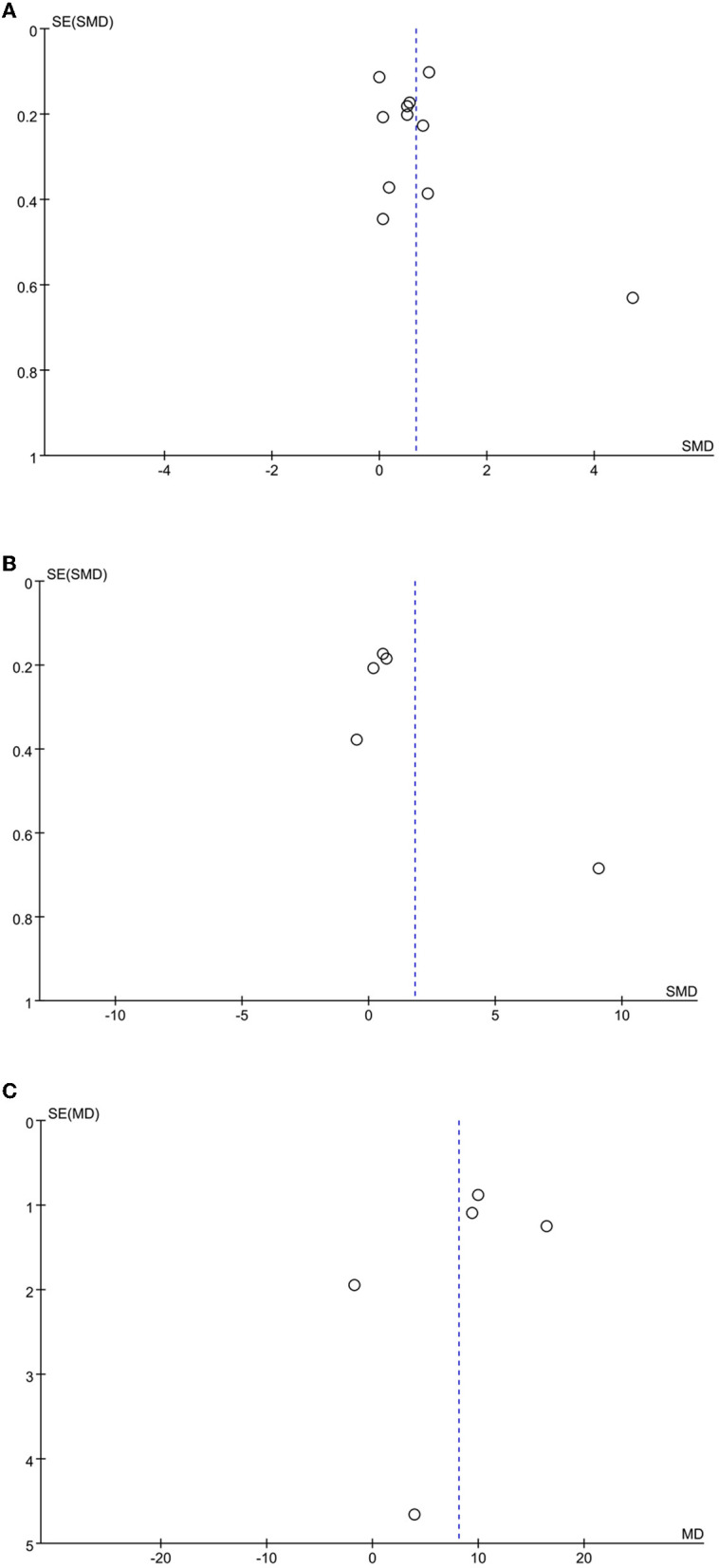
Funnel plot of the included studies. **(A)** cognitive function; **(B)** motor function; **(C)** activities of daily living.

A funnel plot analysis of the included literature with cognitive function, motor function, and activities of daily living as the outcome indicators showed that the distribution was generally symmetric, and the meta-analysis results were reliable (Figure 7).

## Discussion

With the understanding of stroke, people pay attention not only to the problems of motion perception caused by stroke but also to the impact of stroke on cognitive function. After a stroke, the overall cognitive function of most patients is in a state of decline due to brain damage (Li et al., 2013). Physical activity has been shown to increase brain neuronutrients, improve cerebrovascular function and cerebral perfusion, reduce stress responses, and increase brain plasticity through synaptogenesis and nerve regeneration (Li et al., 2013). The 2019 Canadian Stroke Best Practice Guidelines recommend that exercise therapy be considered an adjunctive treatment for cognitive impairment, including attention, memory, and executive performance (Lanctot et al., 2020). In this study, we analyzed two current mainstream scales for comprehensive cognitive assessment and found that the cognitive function score in the exercise therapy group was higher than those in the control group, and the difference was statistically significant (*P* < 0.05). This study strongly demonstrated that exercise therapy can improve cognitive function in patients with PSCI, which is consistent with the findings of Ravichandran et al. (2020). Exercise therapy may improve PSCI for several reasons. Several studies have suggested that exercise can improve cognitive impairment in patients with mild PSCI, possibly because it can improve patients' cardiopulmonary function, reduce brain atrophy volume, increase cerebral blood flow, promote the establishment of brain neural networks, improve brain tissue metabolism, and stimulate central nervous system excitation (Szulc-Lerch et al., 2018). Moreover, exercise can also reduce or delay the occurrence of stroke risk factors such as coronary atherosclerotic heart disease, type 2 diabetes, hypertension, and other common diseases (Callisaya and Nosaka, 2017). A study found that exercise can improve cognitive performance, specifically memory and executive functions, and this was accompanied by an increase in plasma brain-derived neurotrophic factor (BDNF) levels (Sungkarat et al., 2018). In addition, exercise, especially aerobic exercise, significantly improved cortical connectivity and thus improved cognitive function in patients with PSCI (Ahlskog et al., 2011), which is consistent with the results of the subgroup analysis in this study. Studies have shown that exercise therapy can be used as a potentially effective technique to improve cognitive function in patients with PSCI.

In addition to cognitive impairment, motor impairment is a common consequence of stroke. Life becomes more difficult for patients with motor impairments who have PSCI. This study has shown that exercise therapy can improve motor function in patients with PSCI. The patients master the correct motor skills due to repeated, regular coordination training during the exercise. The movement of the cerebral cortex motor area is “set” through the input of repeated and intensified normal movement mode to the brain for stimulation so that the patient's body movements achieve maximum coordination and randomness and then promote the recovery of the affected limb movement ability and effectively reduce the occurrence of hemiplegic limb disuse and misuse atrophy deformation (Li et al., 2016). Many molecular signaling pathways are involved in this process, but among them, the brain-derived neurotrophic factor is a key promoter of neuroplasticity involved in motor learning and rehabilitation after stroke. Exercise, especially aerobic exercise, can upregulate neuronutrients (such as BDNF) to enhance the plasticity of the motor system (Mang et al., 2013). At the same time, exercise therapy can train the limbs of patients with PSCI, improve the condition of their movement impairment, and make their activities more coordinated.

Studies have shown that exercise can improve patients' ability to undertake the activities of daily living and prevent the occurrence of falls in elderly people, especially exercise to maintain physical balance (Sherrington et al., 2017), which is consistent with the findings of this study. García-Rudolph et al. (2019) also found that physical activity can improve the quality of life after a stroke. Exercise promotes the recovery of motor function and improves the activities of daily living. Improving upper extremity function can improve the ability to eat, dress, and use utensils; rehabilitating the back, waist, and lower extremities can promote the rehabilitation of turning, sitting, and standing transfer abilities. Improvements in balance and gait can improve patients' ability to walk and climb stairs (Shiraishi et al., 2017). Therefore, medical staff can improve the activities of daily living in patients with stroke by strengthening exercise therapy.

### Strengths and limitations

The main advantage of this meta-analysis review is that only RCTs were selected. RCTs have the highest level of research evidence, and most high-quality clinical trials use the RCT design method. At present, there are still limitations to this study. First, although we used a rigorous method to search and select literature, publication bias was inevitable because the eligible studies included were Chinese and English literature only. In addition, many Chinese studies on blind methods, allocation, and concealment provided insufficient information, which was a hidden danger. Second, since the study did not strictly screen patients for the time of onset and diagnosis of PSCI, our results may be influenced by inevitable heterogeneity. Third, due to the limited number of eligible studies, no further subgroup analysis of the effects of exercise duration was performed in this study, which could be considered more carefully in subsequent studies.

## Conclusion

In conclusion, exercise therapy can not only significantly improve the cognitive function of patients with PSCI but also improve the motor function and activities of daily living of patients to some extent. However, as the intensity and frequency of exercise therapy are still heterogeneous, the intensity and frequency of exercise therapy should be further discussed. In addition, if exercise therapy has the advantages of feasibility, economy, and safety to improve the quality of life of patients with PSCI, it is worth exploring how it might be promoted further.

## Data availability statement

The raw data supporting the conclusions of this article will be made available by the authors, without undue reservation.

## Author contributions

YZ, XQ, JC, CJ, FW, and DS contributed to the study's conception and design. Material preparation, data collection, and analysis were performed by YZ, XQ, CJ, and CL under the supervision of LC and PY. The first draft of the manuscript was written by YZ, XQ, and JC. All authors commented on previous versions of the manuscript. All authors read and approved the final manuscript.
